# Prolonged neuromuscular blockade and insufficient reversal after sugammadex administration in cesarean section under general anesthesia: a case report

**DOI:** 10.1186/s40981-019-0248-8

**Published:** 2019-04-11

**Authors:** Kuniaki Moriwaki, Kenji Kayashima

**Affiliations:** Department of Anesthesia, Japan Community Health Care Organization, Kyushu Hospital, 1-8-1 Kishinoura, Yahatanishi-ku, Kitakyushu City, Fukuoka, 806-8501 Japan

**Keywords:** Pregnancy, Cesarean section, General anesthesia, Neuromuscular blockade

## Abstract

**Background:**

We present a rare case of prolonged neuromuscular blockade and insufficient reversal after sugammadex administration in a pregnant patient being treated with magnesium sulfate and nifedipine undergoing cesarean section under general anesthesia.

**Case presentation:**

A 37-year-old woman at 34 weeks gestation, weighing 42.5 kg, and receiving magnesium sulfate 94 mg/kg for preeclampsia and nifedipine 20 mg, underwent cesarean section under general anesthesia for abruptio placentae. Her trachea was intubated after administering rocuronium 0.94 mg/kg. Postoperatively, sugammadex 4.7 mg/kg was administered at post-tetanic count 2, 163 min after rocuronium administration. However, 9 min after sugammadex administration, the train-of-four ratio only reached 0.7. Fifteen min after sugammadex administration, extubation was successfully performed when the train-of-four ratio reached 0.9 after administration of atropine 0.5 mg and neostigmine 1.0 mg.

**Conclusions:**

Caution is required in pregnant women on high-dose magnesium sulfate with nifedipine, which may cause prolongation of neuromuscular blockade and insufficient reversal.

## Background

Rocuronium, a neuromuscular blocking agent, provides excellent to good intubating conditions in Cesarean section (C-section) patients, and is safe for both the mother and the fetus [1]. Rapid injection of an intravenous bolus of magnesium sulfate 60 mg/kg administered before rocuronium was shown to significantly prolong recovery from neuromuscular blockade [2]. In the experimental setting, administration of a high dose of magnesium 87 mg/kg caused the twitch height to decrease to 33% of the baseline value [3]. Data on onset of rocuronium action with magnesium are available in non-pregnant [4], but not in pregnant patients.

Sugammadex provides rapid and dose-dependent reversal of profound neuromuscular blockade induced by high-dose rocuronium (1.0 or 1.2 mg/kg) in adult surgical patients [5]. Pre-treatment with magnesium sulfate 40 mg/kg was observed to not significantly affect the recovery time of moderate neuromuscular blockade facilitated by sugammadex [6]. Combination of magnesium sulfate with a calcium-antagonist in patients undergoing C-section under general anesthesia may or may not prolong the neuromuscular blockade [7, 8].

We present here a case of prolonged neuromuscular blockade and insufficient reversal after sugammadex administration in a non-obese pregnant patient being treated with a high-loading dose of magnesium sulfate 94 mg/kg and nifedipine, a calcium-antagonist, who underwent C-section under general anesthesia.

## Case presentation

A 37-year-old woman, at 34 weeks of pregnancy (144 cm height; 42.5 kg body weight), and body mass index (BMI) 20.5 kg/m^2^, underwent C-section under general anesthesia for abruptio placentae. Written informed consent was obtained for publication of the case. The patient had associated gestational diabetes; preeclampsia, treated with magnesium sulfate maintenance infusion at 1 g/h after loading infusion at 4 g/20 min; and hypertension, which was being treated with oral nifedipine 20 mg, the last dose of which was taken 5 h before anesthesia induction. Magnesium sulfate infusion was stopped 10 min before anesthesia induction. Its serum concentration was 4.4 mg/dl, measured about 6 h before anesthesia induction, which was within the target concentration for preeclampsia (4.2–8.4 mg/dl [9]) during maintenance of infusion. Before anesthesia induction, a neuromuscular transmission mechanosensor incorporated in the neuromuscular transmission module (Datex-Ohmeda S/5, GE Healthcare, Tokyo, Japan) was attached to the tips of the left thumb and left index finger; electrodes were attached for stimulating the left ulnar nerve. Neuromuscular monitoring was started with automatic calibration before rocuronium administration. Before rocuronium administration, the train-of-four (TOF) ratio was 1.0. General anesthesia was induced with propofol 100 mg and rocuronium 40 mg (0.94 mg/kg in actual body weight). Laryngoscopy yielded Cormack and Lehane grade 1 view, and the trachea was intubated 40 s after rocuronium administration (TOF count 0). Anesthesia was maintained with sevoflurane 1.0–1.5%, remifentanil 0.1–0.2 mcg/kg/min, and fentanyl 200 mcg, without maintenance with rocuronium. The post-tetanic count (PTC) was 0 postoperatively, 95 min after the induction dose of rocuronium was administered. PTC was performed after an interval of at least 2 min on the monitoring setting. We also used another neuromuscular monitoring method for comparison to detect monitoring failure: the accelerometer of a TOF Watch® (Nippon Koden Co., Tokyo, Japan) attached to the tip of the right thumb with a hand adaptor; electrodes were attached for stimulating the right ulnar nerve. We could not calibrate the TOF Watch®, as there were no muscle contractions. Sugammadex 200 mg (4.7 mg/kg in actual body weight) was administered when PTC 2 was confirmed, 163 min after administration of the induction dose of rocuronium. Atropine 0.5 mg and neostigmine 1.0 mg were administered when the TOF ratio was 0.7, 9 min after sugammadex administration. TOF ratio exceeded 0.9 at 15 min after sugammadex administration. Sevoflurane was turned off 5 min after atropine and neostigmine administration, and the trachea was extubated. The patient recovered uneventfully.

## Discussion

In our case, prolonged neuromuscular blockade and insufficient reversal of neuromuscular blockade were probably due to ongoing treatment with a high loading dose of magnesium 94 mg/kg, or the combination of magnesium sulfate with a calcium antagonist.

The trachea was intubated 40 s after rocuronium administration, which is a short interval. In the emergency department setting, the mean time to onset of paralysis was observed to be 45 s with a mean rocuronium dose of 1.0 mg/kg [10]. Prior administration of magnesium sulfate in non-pregnant patients did not increase the speed of onset of action of rocuronium in a study [2], but reduced the onset time in another [11].

The incidence of failed intubation in pregnant patients has been reported to be high (1 in 300) [12]. Good intubating conditions after rocuronium 0.6 mg/kg administration are not always achieved in patients undergoing C-section under general anesthesia [1, 13]. Rocuronium bromide 1 mg/kg can be safely used for rapid sequential induction in C-sections [14]. The correct dose of rocuronium for rapid sequential induction in an obstetric patient has been suggested as 1.0 mg/kg [15]. The lower the BMI, the shorter the duration of recovery from rocuronium-induced blockade at the initial and additional doses determined by the actual body weight in adult patients with BMI less than 25  kg/ m^2^ [16]. Thus, administration of rocuronium at 0.94 mg/kg seemed appropriate in our patient with a BMI of 20.5 kg/m^2^.

Rocuronium-induced neuromuscular blockade is known to last about 1.1 times (45.7 min vs 40.6 min) longer in women in their second trimester compared to non-pregnant women [17]. Rocuronium 0.9–1.2 mg/kg has a duration of action of approximately 60 min, which is acceptable for C-section [11]. However, in our patient, in whom rocuronium 0.94 mg/kg was administered, the time to achieve PTC 2 was 163 min, which may not be acceptable for a surgical time of 95 min. In patients with diseases such as cirrhosis [18] or renal failure [19], recovery from rocuronium-induced blockade is prolonged; however, our patient did not have any of these conditions.

Magnesium sulfate has been shown to prolong the total time to recovery from a rocuronium-induced blockade by about 25% [11] and 60% [14] in non-pregnant patients. Magnesium sulfate 30 mg/kg accelerated the onset and improved operating conditions of low-dose rocuronium without prolongation of action [4]. Pre-treatment with magnesium sulfate 40 mg/kg did not significantly affect the recovery time of moderate neuromuscular blockade facilitated by sugammadex [6]. Rapid administration of an intravenous bolus of magnesium sulfate 60 mg/kg injected before rocuronium had no influence on the speed of onset but significantly prolonged the neuromuscular block [2]. In the experimental setting, administration of magnesium 87 mg/kg caused the twitch height to decrease to 33% of the baseline value [3]. Loading infusion of magnesium sulfate 94 mg/kg in our patient could have led to prolonged time to recovery from the neuromuscular block.

Calcium-antagonists can potentiate the toxicity of magnesium considerably [20], which may enhance the effects of muscle relaxants [21]. By contrast, one study reported no clinically significant interaction between magnesium sulfate and calcium-antagonists among women with suspected preeclampsia, particularly with respect to neuromuscular weakness or other serious magnesium sulfate-related adverse effects [8]. In another study, in a woman receiving magnesium sulfate and a calcium-antagonist and undergoing C-section under general anesthesia, prolonged and deep neuromuscular blockade induced by vecuronium (6 mg) was antagonized 3 h later with neostigmine [7].

Sufficiently large doses of sugammadex (more than about 1 mg/kg) eliminate the possibility of muscle relaxation rebound [22]. However, in a case report of an obese pregnant patient, sugammadex 2.3 mg/kg failed to reverse neuromuscular blockade [23]. Pre-treatment with magnesium sulfate did not significantly affect the time for a sugammadex-mediated reversal of moderate blockade induced by rocuronium [6]. However, after raising the magnesium concentration of the buffer by 3.5 to 4.1 mmol/L (resulting in a decrease of twitch height to 65% of baseline values), much higher concentrations of sugammadex were required to reverse the block [3]. Reversal of rocuronium-induced blockade by sugammadex is influenced by the degree of the residual blockade at the time the reversal drug is administered [24]. TOF ratio over 0.9 was achieved finally 15 min after administration of sufficiently large doses of sugammadex in our patient because higher rocuronium levels might have been present in the circulating blood and other compartments. We chose atropine and neostigmine because we had already administered sufficiently large doses of sugammadex and the TOF ratio was 0.7 [25].

Succinylcholine remains the neuromuscular blocking drug of choice for obstetric anesthesia because of its rapid onset and short duration of action [26]. Recently, it has been suggested that the use of a combination of rocuronium and sugammadex can increase the safety and quality of care in obstetric patients; thus, the use of succinylcholine in C-sections might no longer be indicated [27].

One of the limitations in our approach to the case was those serum concentrations of rocuronium and magnesium sulfate was not measured during the prolonged blockade; the concentrations of the drugs could have played an important role in the prolongation of neuromuscular blockade.

In conclusion, caution must be exercised when administering high doses of magnesium sulfate treatment relative to body weights in combination with a calcium-antagonist, as it may prolong neuromuscular blockade and cause an insufficient reversal in pregnant women.

## Abbreviations

BMI: Body mass index
C-section: Cesarean section
PTC: Post-tetanic count
TOF: Train-of-four
